# Effectiveness of oral prednisone tapering following intravenous methylprednisolone for acute optic neuritis in multiple sclerosis

**DOI:** 10.1371/journal.pone.0288366

**Published:** 2023-12-07

**Authors:** Adi Wilf-Yarkoni, Kristina Feldmann, Kerstin Rubarth, Eva-Maria Dorsch, Rebekka Rust, Ilia Urman, Mark A. Hellmann, Yitzhak Friedman, Itay Lotan, Omer Bialer, Gilberto Solorza Buenrostro, Hanna G. Zimmermann, Carla Leutloff, Tanja Schmitz-Hübsch, Friedemann Paul, Susanna Asseyer, Hadas Stiebel-Kalish

**Affiliations:** 1 Neuroimmunology Unit, Department of Neurology, Rabin Medical Center, Petah Tikva, Israel; 2 Sackler School of Medicine, Tel Aviv University, Tel Aviv, Israel; 3 Experimental and Clinical Research Center, a Cooperation Between the Max Delbrück Center for Molecular Medicine in the Helmholtz Association and Charité Universitätsmedizin, Berlin, Germany; 4 Charité –Universitätsmedizin Berlin, Corporate Member of Freie Universität Berlin and Humboldt-Universität zu Berlin, Experimental and Clinical Research Center, Berlin, Germany; 5 Max Delbrück Center for Molecular Medicine in the Helmholtz Association (MDC), Berlin, Germany; 6 Neuroscience Clinical Research Center, Charité –Universitätsmedizin Berlin, Corporate Member of Freie Universität Berlin and Humboldt-Universität zu Berlin, Berlin, Germany; 7 Department of Emergency and Acute Medicine, Campus Mitte and Virchow, Charité –Universitätsmedizin Berlin, Berlin, Germany; 8 Charité Universitaetsmedizin Berlin, Corporate Member of Freie Universität Berlin and Humboldt Universität zu Berlin, Institute of Biometry and Clinical Epidemiology, Berlin, Germany; 9 Berlin Institute of Health (BIH), Berlin, Germany; 10 Felsenstein Medical Research Center, Tel Aviv University, Tel Aviv, Israel; 11 Neuro-Ophthalmology Unit, Department of Ophthalmology, Rabin Medical Center, Petah Tikva, Israel; Heinrich-Heine-Universitat Dusseldorf, GERMANY

## Abstract

Acute optic neuritis treatment lacks standardized protocols. The value of oral prednisone taper (OPT) following intravenous methylprednisolone (IVMP) on visual outcome parameters in optic neuritis (ON) has never been explored. In the present retrospective study, we investigated whether OPT after IVMP affects the structural and functional visual outcomes of inaugural clinically isolated syndrome (CIS)- or multiple sclerosis (MS)-ON. Adult patients with acute, inaugural, unilateral CIS- or MS-ON, treated with IVMP in Germany and Israel were stratified into patients treated with IVMP alone—versus IVMP and OPT. Inclusion criteria were age ≥18, CIS or MS diagnosis according to McDonald criteria 2017, available visual acuity (VA) at nadir before treatment initiation and at follow-up ≥5 months, as well as a spectral domain optic coherence tomography (OCT) data scan at follow-up. Exclusion criteria included recurrent ON, concomitant ophthalmological comorbidities, optical coherence tomography (OCT) of insufficient quality and ON-related escalation therapy after IVMP. The structural outcome was defined as the average retinal nerve fiber layer (RNFL) difference between the ON-affected and the unaffected eye, while the functional outcome was defined as the final high-contrast best-corrected VA (HC-BCVA) at follow-up compared to nadir. The comparative analysis was performed using linear regression analysis, adjusted for sex, age, and days-to-treatment. Fifty-one patients met the inclusion criteria (25% male). The mean age was 33.9 (±10.23) years. Twenty-six patients (51%) received OPT following IVMP. There was no difference in nadir HC-BCVA between the groups (0.39 No OPT; 0.49 With OPT, *P* = 0.36). Adjusted linear regression analysis did not indicate an influence of OPT on RNFL thickness or on HC-BCVA (beta coefficient for RNFL difference in percentages: 0.51, 95%-CI: [-4.58, 5.59], beta coefficient for logMAR: 0.11, 95%; CI [-0.12, 0.35] at follow-up. In conclusion, the addition of OPT to IVMP did not affect RNFL thickness or the final VA in a retrospective cohort of 51 patients with inaugural acute CIS- or MS-ON. The results of this exploratory study are currently being re-examined in a large-scale, demographically diverse, prospective study.

## Introduction

Optic neuritis (ON) is an acute inflammatory demyelinating disorder of the optic nerve. ON is a common symptom of clinically isolated syndrome (CIS) and multiple sclerosis (MS) [1]. Current treatment regimens for acute ON in CIS and MS are based on the landmark 1992 Optic Neuritis Treatment Trial (ONTT) [2]. This trial demonstrated that a 3-day course of intravenous methylprednisolone (IVMP) with an additional course of oral prednisone with a rapid taper (OPT) hastened recovery compared to either a course of 1mg/kg oral prednisone with OPT or placebo. Since then, studies have confirmed that IVMP hastens visual recovery after ON, without improving the final visual outcome [3]. Recent studies have questioned the superiority of IVMP to a bioequivalent dose of oral prednisone treatment [4–7], yet the beneficial effect of adding an OPT to IVMP has not been studied despite its widespread use. A retrospective study by Perumal et al. suggests that OPT following IVMP fails to improve disability or speed of recovery from relapses in MS at 12 months follow-up [8]. Bazi [9] and Zeca [10] performed two placebo-controlled trials showing that IVMP plus OPT was neither superior to IVMP alone in treating non-ON MS relapses, nor in preventing adrenal insufficiency. However, the additional steroid dose with OPT was associated with more side effects [9, 10]. None of these studies explored the effect of OPT after IVMP on visual outcomes following acute ON. The aim of our study was to compare whether adding OPT after IVMP has a beneficial effect compared to IVMP alone when treating acute ON in CIS and MS, using structural and functional visual outcomes.

## Methods

### Patients

We performed a retrospective study of patients with acute ON associated with CIS or MS, followed between 2011–2021 at two tertiary referral neuroimmunology centers in Germany and Israel. Both studies adhered to the declaration of Helsinki and were approved by independent medical ethical committees. The German cohort comprised patients who were enrolled in one of two prospective, observational cohort studies (CIS study: ClinicalTrials.gov identifier: NCT01371071, and VIMS study, EA1/182/10) at the Experimental and Clinical Research Center (ECRC) at Charité –Universitätsmedizin Berlin, Germany. The Israeli cohort included patients who were followed by the neuro-ophthalmology and neuroimmunology clinics at Rabin Medical Center, Israel. The study protocol was approved by the Rabin Medical Center Institutional Review Board, with a waiver of informed consent due to the retrospective nature of the study (Helsinki Committee, No. 0152–22).

Inclusion criteria were age ≥18, diagnosis of a first-ever episode of unilateral acute ON and a subsequent diagnosis of CIS or MS, treatment with IVMP, complete ophthalmological assessment including available high-contrast best corrected visual acuity (HC-BCVA) at nadir before treatment initiation and at follow-up ≥5 months, as well as a spectral domain optic coherence tomography (OCT) data scan at follow-up. CIS and MS patients were diagnosed according to the 2017 McDonald diagnostic criteria [11]. Myelin oligodendrocyte glycoprotein (MOG)- and aquaporin-4 (AQP4)-IgG status was tested in patients with atypical ON presentation as it has previously been recommended and emphasized by the International MOGAD Panel proposed criteria [12]. All patients had a confirmed diagnosis of ON [1]. Diagnostic parameters included: a history of painful vision loss, a relative afferent pupil defect in the affected eye, a visual field defect consistent with the diagnosis of ON and a fundus examination showing normal or mild optic disc swelling with ophthalmoscopy with paraclinical biomarker confirmation (visual evoked potentials (VEP) and/or magnetic resonance imaging (MRI) of the brain and optic nerves). Exclusion criteria consisted of a diagnosis of neuromyelitis optica spectrum disorder (NMOSD) or MOG-IgG associated disease (MOGAD), recurrent ON occurring between the inaugural event and follow-up, ophthalmologic comorbidities potentially affecting the OCT, insufficient OCT quality (based on the OSCAR-IB criteria [13], availability of only time-domain OCTs, and treatment with acute ON escalation therapy (including second dose of IVMP, intravenous immunoglobulins (IVIG) and/or plasma exchange)).

Data recorded at nadir were biological sex, age at onset, HC-BCVA (documented as logMAR equivalent), time- lapse from symptoms onset to the beginning of IVMP, and acute steroid regimen. Data collected at follow-up included HC-BCVA and OCT parameters (i.e., average retinal nerve fiber layer (RNFL) thickness (measured in microns) for both the affected and unaffected eye).

### Visual acuity measurement

HC-BCVA was measured during the acute phase of ON at nadir before treatment within the hospital setting, as part of the clinical routine. HC-BCVA was examined by trained ophthalmologists using *Early Treatment Diabetic Retinopathy Study* (EDTRS) charts and given as a decimal value, which was converted into a *Logarithm of the Minimal Angle of Resolution* (LogMAR) units. At follow-up, HC-BCVA was performed at the ECRC, Berlin, using EDTRS charts and converted into LogMAR units. For the Israeli cohort, HC-BCVA was measured by trained ophthalmologists using Snellen charts and converted into LogMAR units.

### Optical coherence tomography

OCT was carried out for the affected and unaffected eyes for all patients at follow-up. In the German cohort, OCT was performed using the Heidelberg Spectralis OCT in the N-Site module. In the Israeli cohort, OCT was performed using the Cirrus HD-OCT (Model 4000, Software V.6.0; Carl Zeiss Meditec), using the Optic Disc 200 × 200 protocol to obtain the average RNFL thickness. At both centers, OCT measurements were carried out by certified ophthalmic technicians. Scans were obtained using a single device, in dim room light, with the patient’s pupils dilated. OCT results are reported in accordance with the APOSTEL 2.0 guidelines [14].

### Study outcome

For analysis, patients were stratified into two groups: those who received IVMP alone (No OPT) compared to patients who received OPT after IVMP treatment (With OPT). Two outcomes were selected for this study; the average RNFL thickness of the affected eye compared to that of the unaffected eye (in percentages), and the HC-BCVA at final follow-up (absolute value and difference from HC-BCVA at presentation).

### Statistics

Descriptive demographic data was analyzed using summary statistics (mean and standard deviation for normally distributed variables, median, first and third quartiles for non-normally distributed variables as well as the absolute and relative frequencies for categorical variables). Patient characteristics were compared using two-sided t-tests for metric data as well as chi-square-tests for categorical data.

To investigate the effect of OPT on RNFL values between the unaffected eye and the affected eye in percentages, a linear regression analysis was conducted, adjusted for sex, age at ON onset, and time from visual loss to treatment (in days). To analyze the impact of OPT on LogMAR differences between nadir and follow-up in the affected eye, a second linear regression analysis was performed, using the same set of predictor variables. Due to the exploratory nature of the study, no adjustment for multiplicity was conducted. Therefore, p-values and confidence intervals are interpreted in an exploratory, hypothesis-generating manner.

We performed an a-priori power analysis to determine the required sample size for our study. We anticipated that the standard deviation of the RNFL differences, measured as a percentage, would be approximately 9%. We considered a clinical significance threshold of an 8% difference in RNFL values. To detect this difference with a two-sample, two-sided t-test at a significance level (alpha) of 5% and a desired statistical power of 80%, we calculated that we would need approximately 21 patients in each group.

## Results

### Patients and demographics

**Fig 1:**
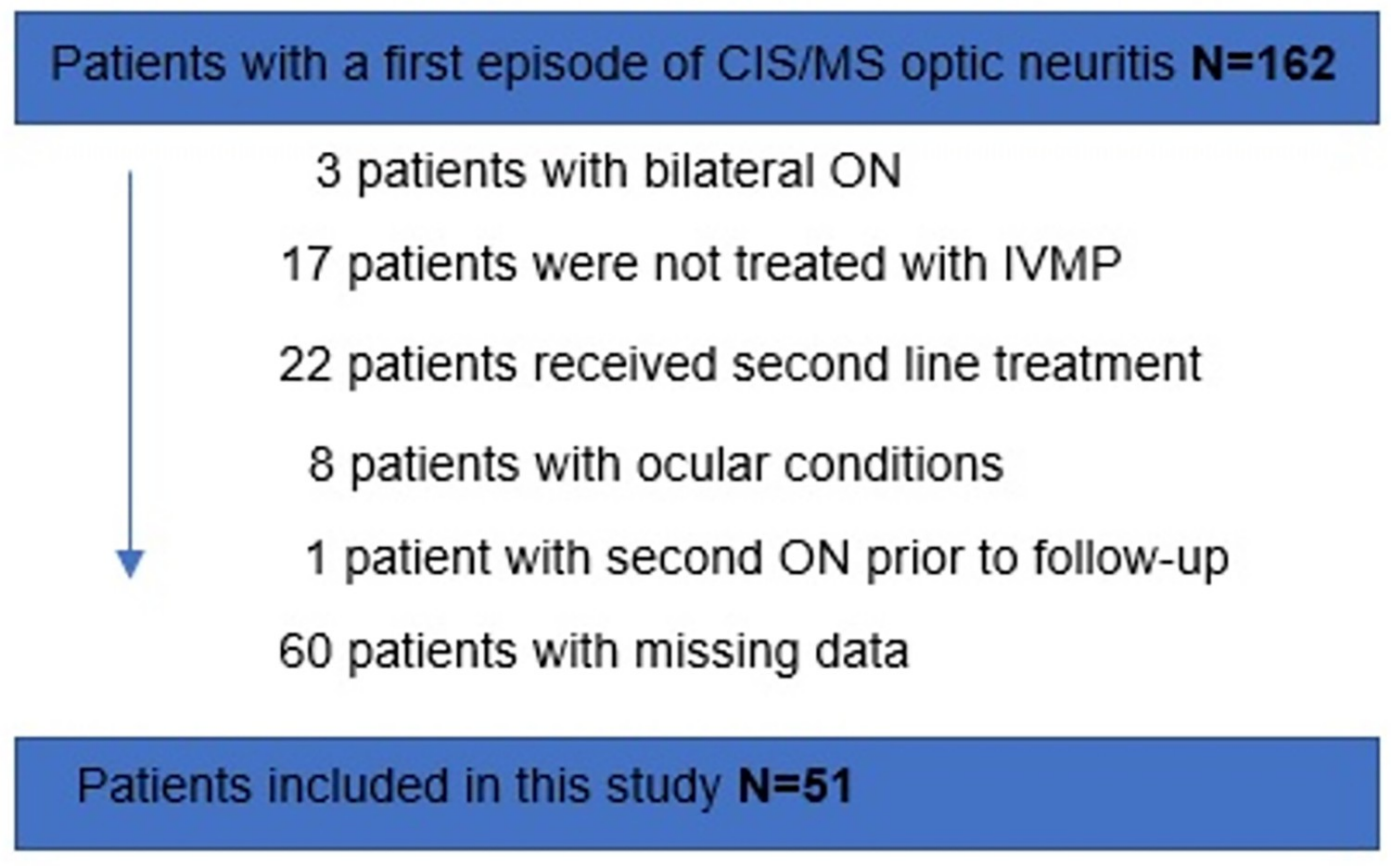
Flow chart. Study flowchart. CIS: clinical isolated syndrome; f/u: follow-up; IVMP: intravenous methylprednisolone; MS: multiple sclerosis; OCT: optical coherence tomography; ON: optic neuritis; VA: visual acuity.

We identified 162 patients with first-ever acute ON who were subsequently diagnosed with CIS or MS (see Fig 1). One-hundred and eleven patients had to be excluded due to bilateral ON (n = 3), absence of IVMP-treatment (n = 17), additional second-line treatment (n = 22), severe ophthalmologic comorbidities (n = 8), optic neuritis relapse between baseline and follow-up (n = 1) or an incomplete dataset (n = 60).

In total, 51 patients met our inclusion criteria. The mean age at onset was 33.96 years (±10.23). Thirteen subjects (25.5%) were males. Mean follow up time was 323 [±107] days. Patients were stratified according to whether they received OPT after IVMP treatment (No OPT n = 25; With OPT n = 26) with a mean age at onset of 31.44 [±8.36] and 36.38 [±11.39] years in each group, respectively (P = 0.08). Clinical and demographic characteristics are summarized in Table 1.

**Table 1:** Demographic and clinical data for patients with optic neuritis.

	Total	No OPT	With OPT	p value
Number of patients (N)	51	25	26	
Sex [male; %]	[13; 25.5]	[8; 32]	[5; 19]	0.469
Age at ON onset in years (mean; [SD])	33.96 [10.23]	31.44 [8.36]	36.38 [11.39]	0.084
Median dose of IVMP in mg (median; [IQR])	5000 [4500, 5000]	5000 [4000, 5000]	5000 [5000, 5000]	0.484
Days from presentation to treatment [SD])	9.47 [13.98]	8.40 [6.80]	10.50 [18.55]	0.597
Days from nadir to follow-up [SD])	323.94 [107.17]	350.28 [112.40]	298.62 [97.37]	0.085

Patients with ON treated without OPT vs. patients treated with IVMP followed by OPT. IQR: Interquartile range; IVMP: intravenous methylprednisolone; N: number; ON: optic neuritis; OPT: oral prednisone taper; SD: Standard Deviation. Accumulative dose of IVMP, given in mg.

### Treatment outcomes

**Fig 2:**
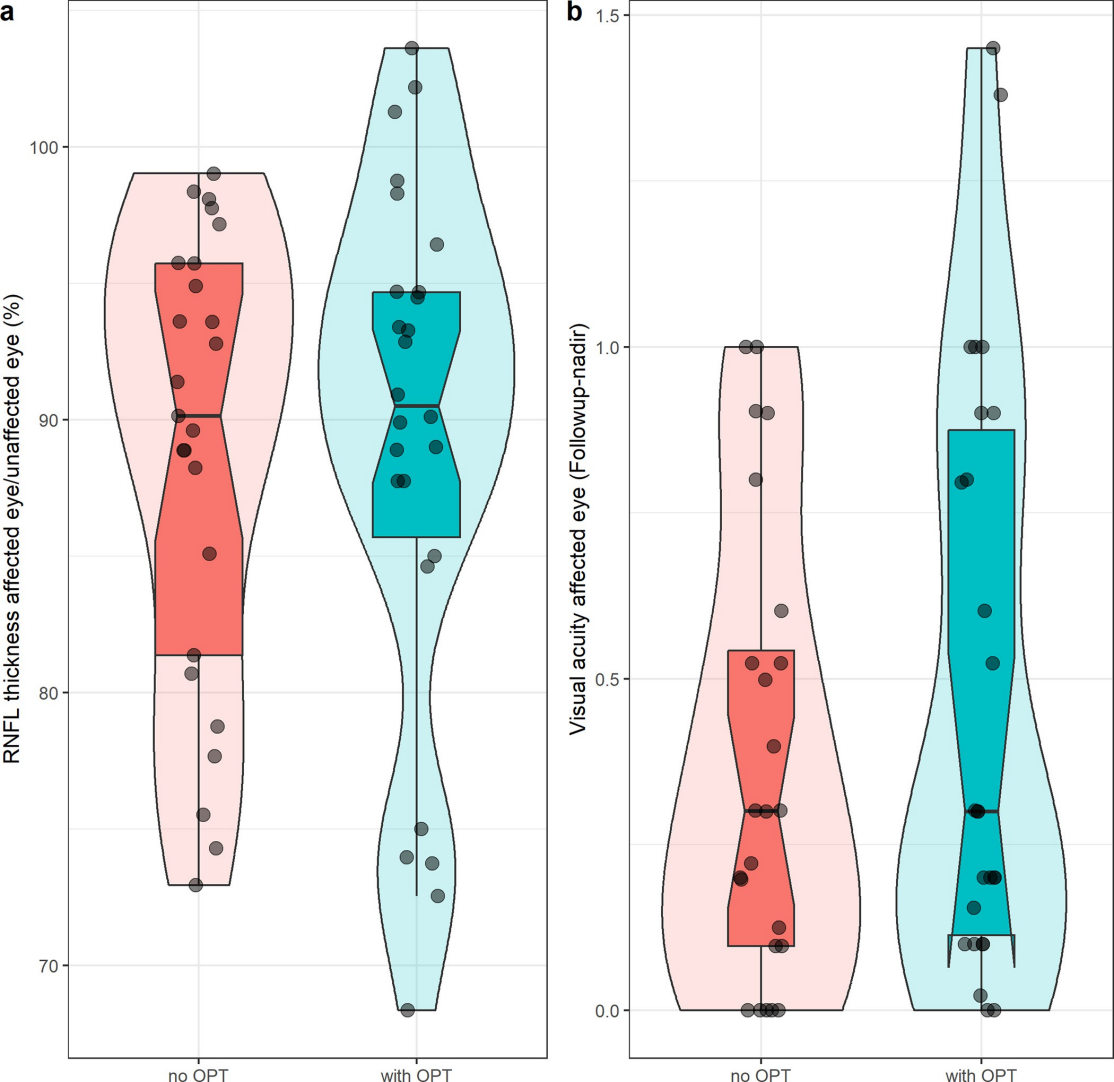
The distribution of average retinal nerve fiber layer (RNFL) thickness (2a) and high-contrast best -corrected acuity (HC-BCVA) (2b) in patients with CIS- or MS optic neuritis. 2a: the average RNFL thickness of the affected eye compared to that of the unaffected eye (in %), and 2b: the HC-BCVA in LogMAR at final follow-up (difference from nadir). Left boxplots, red: patients treated with intravenous methylprednisolone without oral prednisone taper (No OPT). Right box plots, blue: patients treated with intravenous methylprednisolone with oral prednisone taper (With OPT). Box plot details: thick horizontal bar: median Box: interquartile range (25%–75%) Whiskers: range. Dots: outliers (data>1.5 times the interquartile range off the box). The violin plot in the background visualizes a smoothened density estimate.

At follow-up of at least 5 months after onset, both groups demonstrated lower RNFL thickness in the affected eye compared to the unaffected eye (No OPT 88.81% ±8.26 vs. With OPT 89.29% ±9.63) (see Table 2 and Fig 2A). However, there was no RNFL difference between the groups (b = 0.51, 95% CI: [-4.58–5.59] p = 0.842) (see Table 3).

**Table 2:** Visual acuity and retinal nerve fiber layer characteristics at nadir and follow-up.

	Total	No OPT	With OPT	p value
HC-BCVA affected eye at nadir (mean; [SD])	0.44 (0.40)	0.39 [0.34]	0.49 [0.45]	0.360
HC-BCVA unaffected eye at nadir (mean; [SD])	0.01 (0.02)	0.01 (0.2)	0.01 (0.03)	1.00
HC-BCVA affected eye at follow-up (mean; [SD])	0.02 (0.05)	0.03 [0.06]	0.01 [0.03]	0.121
HC-BCVA unaffected eye at follow-up (mean; [SD])	0.01 (0.03)	0.01 [0.02]	0.01 [0.03]	0.276
HC-BCVA difference affected eye follow-up-nadir (mean; [SD])	0.43 (0.40)	0.37 (0.34)	0.49 (0.44)	0.326
HC-BCVA difference unaffected eye follow-up-nadir (mean; [SD])	0.00 (0.03)	0.00 (0.03)	0.00 (0.03)	0.389
HC-BVA difference affected to unaffected eye at follow-up (mean; [SD])	0.01 (0.05)	0.02 (0.06)	0.00 (0.03)	0.335
RNFL affected eye at follow-up (mean; [SD])	86.00 (15.03)	89.48 [15.63]	82.65 [13.90]	0.105
RNFL unaffected eye at follow-up (mean; [SD])	96.57 (13.71)	100.48 [12.91]	92.81 [13.64]	0.045
RNFL difference (mean; [SD])	10.57 (8.68)	11.00 [8.03]	10.15 [9.41]	0.732
RNFL difference in percentage (mean %; [SD])	89.05 (8.89)	88.81 [8.26]	89.29 [9.63]	0.850

Patients with ON treated without OPT (No OCT) vs. patients treated with IVMP followed by OPT (With OCT). HC-BCVA: High contrast best corrected visual acuity in LogMAR. OPT: oral prednisone taper. RNFL: retinal nerve fiber layer thickness in μm measured using optical coherence tomography (OCT); SD: Standard Deviation.

**Table 3:** Linear regression model analysis for the retinal nerve fiber layer in the optic neuritis-affected eye compared to the unaffected eye (in %).

	RNFL in percentage		
Predictors	regression coefficient b	95%-Confidence interval	p value
(Intercept)	96.22	87.36–105.08	<0.001
Gender [Male]	-6.09	-11.78 –-0.39	0.037
Age at ON-Onset	-0.19	-0.44–0.06	0.129
Time to treatment	0.07	-0.11–0.25	0.435
OPT	0.51	-4.58–5.59	0.842

Observations n = 51. R2/R2 adjusted 0.129/0.053. OCT: optical coherence tomography. ON: Optic neuritis. OPT: Oral prednisone tapering. RNFL: retinal nerve fiber layer.

HC-BCVA of the affected eye at nadir was similar in patients treated with either IVMP alone and in patients treated with OPT following IVMP (0.39 ±0.34 logMAR vs. 0.49 ±0.45 logMAR respectively, p<0.36) (see Table 2 and Fig 2B). As an indicator for recovery of visual acuity over time, we compared HC-BCVA between the affected and the unaffected eye at follow-up. In all 51 patients, HC-BCVA of the affected eye recovered to nearly match the HC-BCVA of the unaffected eye (total mean -0.01 [SD +-0.05] logMAR; No OPT mean -0.02 [SD +-0.06] logMAR; With OPT mean -0.00 [SD +-0.03] logMAR). OPT did not affect the recovery of VA compared to patients who did not receive a prednisone taper (b = 0.11, 95%CI: [-0.12–0.35] p = 0.332) (see Table 4).

**Table 4:** Linear regression model of visual acuity differences of affected eye from nadir to follow-up.

	HC-BCVA difference		
Predictors	regression coefficient b	95%-Confidence interval	p value
(Intercept)	0.51	0.09–0.92	0.018
Gender [Male]	-0.10	-0.36–0.16	0.454
Age at ON Onset	-0.00	-0.01–0.01	0.774
Time to treatment	-0.01	-0.01–0.00	0.205
OPT	0.11	-0.12–0.35	0.332

Observations n = 51. R2/R2 adjusted 0.073/-0.010. HC-BCVA: high-contrast best corrected visual acuity, documented as logMAR equivalent. ON: Optic neuritis. OPT: Oral prednisone tapering.

Age at onset did not have an impact on RNFL thickness (b = -0.19, 95% CI: [-0.44, 0.06], p = 0.129) or on HC-BCVA recovery (b = 0.00, 95% CI [-0.01–0.01], p = 0.774). Male sex was the only parameter associated with a worse outcome in terms of RNFL thickness (b = -6.08, 95% CI: [-11.78, -0.39], p = 0.037) but not for HC-BCVA outcome (See Tables 3 and 4).

## Discussion

Our study is the first to analyze the effect of OPT following IVMP on RNFL and visual acuity in CIS- and MS-related inaugural ON events. In our study, the use of OPT following IVMP did not have an additive effect on RNFL thickness when compared to IVMP alone. Furthermore, VA at follow-up was similar in both treatment groups.

There is limited information comparing the optimal regimen of corticosteroids for the treatment of MS attacks [15–18]. Since the ONTT study, OPT following IVMP became common practice, although the additional oral taper was not compared to IVMP alone [2]. Our results complement two previous prospective, placebo-controlled studies and a retrospective study which demonstrated that OPT following IVMP does not improve final clinical outcome in non-ON MS relapses compared to IVMP alone [8–10]. In our study, we chose not to compare the expanded disability status scale (EDSS) but rather RNFL thickness, since RNFL thickness as an outcome is potentially more sensitive in detecting changes over time than the EDSS which is a composite of numerous functional outcomes, and thus more prone to averaging artefacts.

Furthermore, MS-ON has excellent visual outcomes, with over 90% of patients recovering an HC-BCVA of 20/40 or better [2, 19]. Thus, HC-BCVA alone is an insufficient indicator of residual deficits after ON. We expect that low-contrast BCVA (LC-BCVA) may prove a better functional outcome measure of similar future prospective studies [20].

The increasing use of OCT in the diagnosis and follow up of ON patients has shown that most individuals with ON sustain neuroaxonal damage despite full recovery of HC-BCVA [21–24]. Raising awareness of frequent ON-related retinal damage makes the RNFL thickness a good outcome parameter for clinical trials [25, 26]. The RENEW study demonstrated that retinal thinning occurred shortly after ON onset [27]. Furthermore, several studies have shown that using the inter-ocular difference in RNFL thickness for assessing retinal damage, as has been done in our study, is a more sensitive approach in inaugural ON patients [21, 28–30]. P100 on VEP may offer a sensitive measurement as emphasized in the RENEW study [27], unfortunately most of our patients did not undergo VEP during the acute phase.

Our study results highlight an important shortcoming of using the inter-ocular difference in RNFL thickness as a major outcome in MS studies. In our cohort, the RNFL of the unaffected eyes of patients receiving OPT seemed to be thinner than those not receiving OPT as a consequence of outlier results from two patients with thin RNFL in their clinically unaffected eye despite detailed data showing these patients did not subjectively experience or recall a prior event. The inter-ocular difference relies heavily on two assumptions; 1) that all ON events are clinically noticed and recalled by the patient, and 2) that the unaffected eye undergoes little to no RNFL thinning as part of the disease. We know well that both these assumptions have been disproven. Many ON events are clinically silent [31, 32] and a process of progressive RNFL thinning in MS without attacks has been demonstrated [31]. Indeed, Davion et al. [33] reported that MRI can detect optic nerve lesions in half of non-ON eyes in subjects with MS and suggested that this finding corresponds to the majority of cases with retinal neuroaxonal loss in clinically non-ON eyes. In our study, the RNFL of the unaffected eyes of patients receiving OPT seemed to be thinner than those not receiving OPT. This significance is a result of outlier results from two patients with thin RNFL in their clinically unaffected eye despite detailed data showing these patients did not subjectively experience or recall a prior event.

Previous MS studies have shown that male patients tend to have worse clinical outcomes, faster disease progression and higher burden of destructive lesions on MRI studies relative to female patients [34, 35]. In the current study, male sex was associated with reduced RNFL regardless of treatment regimen. Our results are in line with recent reports demonstrating sex difference in RNFL after acute ON [36]. OPT following IVMP treatment is associated with more side effects such as increased appetite, weight gain, mood disorder, and hyperglycemia [9, 10]. Although hypothalamic-pituitary axis inhibition was not evaluated in our study, it has already been shown that abrupt discontinuation of high-dose IVMP does not suppress the pituitary-hypothalamic axis in patients with MS relapses [10, 37]. Thus, there is no physiological need for an oral prednisone taper.

There are several limitations of this study. These include the relatively small sample size and the retrospective design of the study. The lack of randomization is another limitation that merits special consideration, because it may represent a bias to treat relatively more severe ON with OPT. However, HC-BCVA at nadir did not differ between the two groups, suggesting no severity bias towards the group of ON patients receiving OPT. The similarity in nadir and final HC-BCVA between the patients treated with OPT following IVMP and those treated with IVMP alone suggests that a lack of randomization did not affect the clinical outcome. Moreover, HC-BCVA alone is an insufficient indicator of residual deficits after ON. Another limitation is that we compared RNFL thickness between the affected and the unaffected eye at follow-up and not to RNFL at baseline as previously discussed. Last, we only included CIS- and MS-ON in the study, since MOG-IgG-and NMOSD-related ON should be treated differently, and steroid tapering is important to avoid ON relapses in antibody-mediated ON.

## Conclusions

Our exploratory study suggests that there is no benefit to structural or visual outcomes when adding oral prednisone taper to IVMP in patients with acute CIS—and MS-related ON. We are currently enrolling patients in a larger prospective study to validate our results using LC-BCVA, VEP, MRI and OCT with the aim to prevent unnecessary steroid use and related side effects [38].
